# Effects of Selenium on Growth and Selenium Content Distribution of Virus-Free Sweet Potato Seedlings in Water Culture

**DOI:** 10.3389/fpls.2022.965649

**Published:** 2022-07-06

**Authors:** Huoyun Chen, Qun Cheng, Qiaoling Chen, Xingzhi Ye, Yong Qu, Weiwu Song, Shah Fahad, Jianhua Gao, Shah Saud, Yi Xu, Yanfen Shen

**Affiliations:** ^1^Academy of Agricultural Sciences, Enshi Tujia and Miao Autonomous Prefecture, Enshi, China; ^2^Hubei Enshi South China Potato Research Center, Enshi, China; ^3^Enshi Comprehensive Test Station of Sweet Potato Industry Technology System, Enshi, China; ^4^Hainan Key Laboratory for Sustainable Utilization of Tropical Bioresource, College of Tropical Crops, Hainan University, Haikou, China; ^5^Department of Agronomy, Faculty of Agricultural Sciences, The University of Haripur, Haripur, Pakistan; ^6^College of Life Science, Linyi University, Linyi, China

**Keywords:** sweet potato, selenium, hydroponic seedlings, selenium distribution, SPAD value, photosynthesis

## Abstract

Understanding the selenium tolerance of different sweet potato [*Dioscorea esculenta* (Lour.) Burkill] is essential for simultaneously for breeding of new selenium-tolerant varieties and improving the selenium content in sweet potato. Therefore, a greenhouse experiment was conducted from February to April 2022 to evaluate the effect of sweet potato cultivars and selenium (Na_2_SeO_3_) concentrations (0–40 mg/L) on plant growth, physiological activities and plant selenium content distribution. The results showed that when the selenium concentration was more than 3 mg/L, the plant growth was significantly affected and the plant height and root length were significantly different compared to the control. While the selenium concentration was 20 and 40 mg/L had the greatest effect on plant growth when the number of internodes and leaves of the plant decreased, the root system stopped growing and the number of internodes of the plant, the number of leaves and the dry-to-fresh weight ratio of the plant a very significant level compared to reached control. The relative amount of chlorophyll in leaves under treatment with a selenium concentration of 1 mg/L was increased, and the relative amount of chlorophyll in 3 mg/L leaves gradually increased with the increase in the selenium concentration. The values of the maximum photochemical efficiency PSII (fv/fm) and the potential activity of PSII (fv/fo) compared to the control under treatment with 40 mg/L selenium concentration and photosynthesis of plants was inhibited. The selenium content in root, stem and leaf increased with the increase in selenium concentration, and the distribution of selenium content in the plant was leaf <stem <root, and the selenium content in root was significantly higher than that in stem and leaf. In summary, the appropriate concentration of selenium tolerance has been determined to be 3 mg/L. The aquatic culture identification method of selenium tolerance of sweet potatoes and growth indices of various selenium tolerant varieties (lines) established in this study will provide a technical basis for selenium tolerant cultivation and mechanism research.

## Introduction

Selenium is a nutrient element of plants. In the process of plant growth, an adequate amount of selenium can relieve stress such as high temperature, ultraviolet, low temperature and drought, inhibit the production of plant active oxygen (Chu et al., 2010; Djanaguiraman et al., 2010; Hasanuzzaman and Fujita, 2011; Yao et al., 2011), affect plant photosynthesis (Zhang et al., 2014). It is believed that selenium can reduce the effects of oxidative stress caused by other factors stimulating the antioxidant system of plants (Hasanuzzaman and Fujita, 2011; Sieprawska et al., 2015; Gupta and Gupta, 2017). The concentration of antioxidant metabolites in tissues of selenium-rich plants was usually higher than that of non-hyper accumulative plants (Freeman et al., 2010).

In recent years, the bioremediation of inorganic forms of selenium for human and animal consumption through plant approaches (White and Broadley, 2009) and phytoremediation (Pilon-Smits, 2005) to improve selenium levels in selenium-rich soils has become a research hotspot. Studies have shown that low levels of selenium play a beneficial role in Tartary buckwheat (Breznik et al., 2005), pumpkin (Germ et al., 2005), tomato (Shu et al., 2010) and sweet potato (Huang et al., 2020). Seleniferous soils are widespread in the Great Plains of the United States, Canada, South America, Australia, India, China, and Russia (Dhillon and Dhillon, 2003; Fordyce, 2013; Barker and Pilbeam, 2015). However, soil selenium is based on the total selenium content of soil, not the soil available selenium content, but rather what plants can absorb and utilize is soil available selenium. The degree of selenium absorption by plants depends on species, and the selenium accumulation ability of different plants species are varies greatly. In recent years, evolutionary biologists have been concerned with the development of non-accumulative plants into selenium-rich plants. In addition, evolutionary biologists have been concerned with the development of non-accumulative plants into selenium-rich plants. In Caryophyllaceae, Asterales, Brassicales, Fabales, Lamiales, and Rubiaceae, selenium hyperaccumulators have evolved independently through the convergence and evolution of appropriate biochemical pathways into the core of Gentianaceae (Cappa et al., 2014; White, 2016, 2017).

Enshi Prefecture is rich in selenium resources. The sweet potato cultivation area in Enshi prefecture is 37900 ha all year round, the cultivation area is extensive, and has good conditions for biological enrichment of selenium (Enshi Statistical Year book, 2019). However, the production of selenium-rich sweet potato is restricted by factors such as the elevation variation, diversity of regional environment, uneven distribution of selenium levels in soil, and the relatively single source of sweet seed potatoes. Therefore, the study of selenium tolerance of sweet potato is of great significance for breeding of new selenium-tolerant varieties and improving the selenium content in sweet potato. Most of our previous studies achieved the purpose of increasing the selenium content of sweet potato by selecting selenium-rich soil or artificially adding selenium-containing fertilizer, and preliminarily studied the content distribution and morphological distribution of selenium in sweet potato. However, the difference of soil selenium composition in different locations will inevitable the accuracy of screening for the ability of sweet potatoes to be rich in selenium. Adding selenium to the nutrient solution of aquatic seedlings can be an effective method to study the ability of plants to be rich in selenium. In recent years, plant tissue culture technology has developed rapidly. The selection of hydroponic and the rapid propagation of test-tube plantlets can overcome the influence of uneven field stress and ensure that the properties of materials can be expressed under relatively consistent conditions. There are many previous studies on the salt stress of sweet potato (Luan et al., 2007; Yan et al., 2016; Luo et al., 2017; Mondal et al., 2022). However, the response of virus-free sweet potato seedlings to high selenium concentrations and screening the index of selenium tolerance of sweet potato to assess the selenium tolerance of sweet potato varieties has not been reported.

Although many studies have focused on how to improve the selenium content of fresh sweet potato and the distribution of selenium content in different parts of the sweet potato, although sweet potato varieties with different selenium-rich ability have been selected, most of the sweet potato varieties have been screened by this method have failed to find out their selenium-rich threshold, so this study uses the method of adding Na_2_SeO_3_ to hydroponic nutrient solution to simulate selenium stress. The effects of different selenium concentrations on the related agronomic characters, photosynthetic characteristics and plant selenium content distribution of sweet potato plants widely planted in two regions were studied to provide technical methods and data reference for early stage screening of selenium-resistant varieties. The purpose of this study is to provide theoretical reference for the screening of selenium-resistant varieties and planting late-stage areas with high selenium levels.

## Materials and Methods

### Experimental Design

#### Plant Materials and Growth Conditions

**FIGURE 1:**
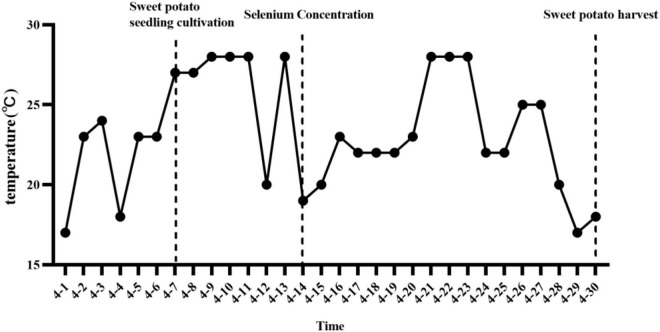
The average daily temperature of the sweet potato hydroponic period in the test area.

The experiment was carried out in the sweet potato hydroponic greenhouse of Enshi Academy of Agricultural Sciences (30°19′ N, 109°28′ E, 430 m a.s.l.) from February to April 2022. The virus-free tissue culture seedlings were removed from the tissue culture flask after refining, the original root was retained, and the residual medium was washed out with pure water. Propagation of virus-free tissue culture seedlings into water-cultured seedlings. The water-cultured seedlings were placed in the greenhouse at a temperature of 10°C∼28°C (Figure 1) for natural light scattering.

#### Sweet Potato Cultivar and Selenium Treatments

On April 7, the virus-free tissue culture seedlings of Shangshu19 and 20167-6 (Large area planting in this area, Table 1) were planted on a special planting plate with a density of 5 cm × 5 cm. 20 sweet potato seedlings of relatively uniform size were transplanted to each plate and placed in a culture plate of 30 cm × 30 cm × 5 cm, and nutrient solution was added to the culture plate to induce the formation of root primitive (Table 2), the root primitive appeared in 5∼7 days. After the formation of the original root body of water-cultured seedlings, the nutrient solution for inducing the original root body was replaced with the rooting solution (Table 3). Rooting nutrient solution was replaced by subculture growth nutrient solution (Table 4). On April 14, the plant was treated with Na_2_SeO_3_ at a concentration of 0 (CK), 0.2, 0.5, 1, 3, 5, 10, 20, and 40 mg/L, and pH regulation (5.8 ± 0.5). The experiment was repeated for three times.

**TABLE 1:** Information of sweet potato varieties.

Variety	Male parent	Female parent	Approved time	Breeder	Breeding unit	Variety characteristics
Shangshu19	Yushu7	sl-01	2003	Shusheng, Lei, Gaimei, Yan, Yuanhua, Li	Shangqiu Institute of Agriculture and Forestry Sciences	The tuber is spindle shaped, with dark red skin and white flesh. The drying rate is 36–38% and the starch content is 23–25%
20167-6	Group hybridization	Xushu27	–	Qun, Cheng, Yi, Xu, Yong, Qu	Academy of Agricultural Sciences of Enshi Tujia and Miao Autonomous Prefecture	The tuber is long spindle shaped, with red skin color and purple flesh color. The sun drying rate is 26–28.79%, and the starch content is 17–20%

**TABLE 2:** Preparation of nutrient solution for inducing root primitive body formation.

Reserve liquid	Composition	Take the amount (mg/L)	Amount of nutrient solution per culture plate (ml)
A	NH_4_NO_3_	16,500	10
	KNO_3_	19,000	
	KH_2_PO_4_	1,700	
	MgSO_4_⋅7H_2_O	3,700	
	CaC_*l2*_	3,330	
B	FeSO_4_⋅7H_2_O	5,570	2
	Na_2_⋅EDTA	7,450	

*When preparing reserve liquid A, finally add calcium chloride; when preparing reserve liquid B, dissolve FeSO_4_⋅7H_2_O and Na_2_⋅EDTA in their respective 450 ml distilled water, heat properly and stir continuously. Then mix the two solutions together, pH 5.5, and finally add distilled water to 1,000 ml. The pH value was adjusted to 5.8 ± 0.5.*

**TABLE 3:** Preparation of rooting nutrient solution.

Reserve liquid	Composition	Take the amount (mg/L)	Amount of nutrient solution per culture plate (ml)
A	NH_4_NO_3_	16,500	50
	KNO_3_	19,000	
	KH_2_PO_4_	1,700	
	MgSO_4_⋅7H_2_O	3,700	
	CaC_*l2*_	3,330	
B	FeSO_4_⋅7H_2_O	5,570	2
	Na_2_⋅EDTA	7,450	
C	KI	830	0.6
	Na_2_MoO_4_⋅2H_2_O	250	
	CuSO_4_⋅5H_2_O	25	
	CoCl_2_⋅6H_2_O	25	
	MnSO_4_⋅4H_2_O	22,300	
	ZnSO_4_⋅7H_2_O	8,600	
	H_3_BO_3_	6,200	

*When preparing reserve liquid A, finally add calcium chloride; when preparing reserve liquid B, dissolve FeSO_4_⋅7H_2_O and Na_2_⋅EDTA in their respective 450 ml distilled water, heat properly and stir continuously. Then mix the two solutions together, pH 5.5, and finally add distilled water to 1,000 ml. The pH value was adjusted to 5.8 ± 0.5.*

**TABLE 4:** Preparation of nutrient solution for subculture and proliferation.

Reserve liquid	Composition	Take the amount (mg/L)	Amount of nutrient solution per culture plate (ml)
A	NH_4_NO_3_	16,500	100
	KNO_3_	19,000	
	KH_2_PO_4_	1,700	
	MgSO_4_⋅7H_2_O	3,700	
	CaC_*l2*_⋅2H_2_O	3,330	
B	FeSO_4_⋅7H_2_O	5,570	6
	Na_2_⋅EDTA	7,450	
C	KI	830	1
	Na_2_MoO_4_⋅2H_2_O	250	
	CuSO_4_⋅5H_2_O	25	
	CoCl_2_⋅6H_2_O	25	
	MnSO_4_⋅4H_2_O	22,300	
	ZnSO_4_⋅7H_2_O	8,600	
	H_3_BO_3_	6,200	

*When preparing reserve liquid A, finally add calcium chloride; when preparing reserve liquid B, dissolve FeSO_4_⋅7H_2_O and Na_2_⋅EDTA in their respective 450 ml distilled water, heat properly and stir continuously. Then mix the two solutions together, pH 5.5, and finally add distilled water to 1,000 ml. The pH value was adjusted to 5.8 ± 0.5.*

### SPAD and Photosynthesis Determination

On April 29, the relative content of chlorophyll (expressed by SPAD value) in the leaves of top 3 seedlings representative plants were measured by SPAD-502 chlorophyll meter, and the measured leaves were marked. The marked leaves were clamped with a shading clamp, and after 30 min dark adaptation, the chlorophyll fluorescence parameters of the corresponding leaves were measured by PAM portable chlorophyll fluorescence meter (Walz, Germany). The initial fluorescence (Fo) under dark adaptation was measured by a weak measuring light (0.12 μmol⋅m^–2^⋅s^–1^, 600 Hz), and then the maximum fluorescence (Fm) under dark adaptation was measured with a saturated pulse light (4,000 μmol⋅m^–2^⋅s^–1^, pulse time 0.8 s). The other parameters were calculated as follows:

Maximum photochemical efficiency of PSII under dark adaptation Fv/Fm = (Fm-Fo)/Fm;

Potential activity of PSII Fv/Fo = (Fm-Fo)/Fo

### Main Agronomic Characters and Selenium Content in Plants

After the determination of SPAD and photosynthetic parameters, 10 plants of each variety (line) were washed three times with distilled water, and the plant height, stem diameter, root length, stem node number and leaf number of each sweet potato seedling were measured, and then the fresh weights of 10 seedlings were quickly dried with absorbent paper and sequentially numbered. The dry matter content was determined by drying weighing method. The numbered seedlings were placed in the oven and dried at 105°C for 30 min and dried at 80°C to weigh the dry weight, respectively.

The dried materials were crushed by a high-speed grinder and screened by 1 mm, then digested into colorless and transparent with a mixed acid (volume of nitric acid: perchloric acid = 4:1). The selenium content was determined by using the atomic fluorescence spectrometer AFS-230 [specific methods refer to ecological geochemical evaluation of animal and plant samples-part 2: determination of the selenium content by atomic fluorescence spectrometry (DZ/T0253.2-2014)].

### Data Analysis

Data analysis uses Microsoft Excel 2010 for data processing, SPSS 23.0 was performed for statistical analyses. A three-way analysis of variance (ANOVA) was used to show the effects of selenium concentrations on plant growth, physiological activities and plant selenium content distribution with Fisher’s Least Significant Difference (LSD) test and Duncan test (Sig < 0.05; Sig < 0.01). Sigma Plot 19.0 version was used to perform the Figures.

## Results and Analysis

### The Growth of Sweet Potato Seedlings in Water Culture

**FIGURE 2:**
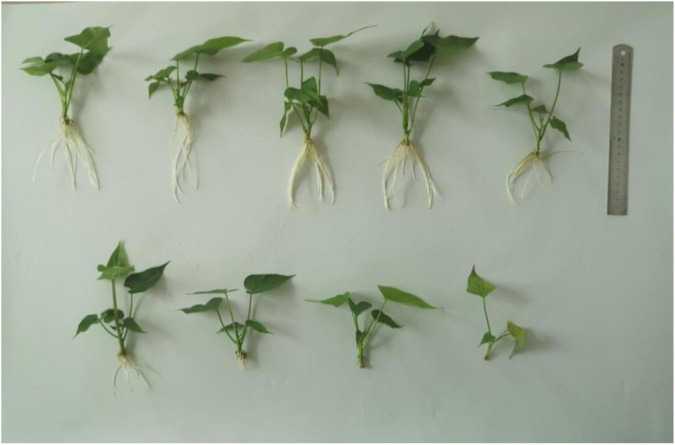
Growth of Shangshu19 under different selenium concentrations. The selenium concentrations in the first row from left to right are 0, 0.2, 0.5, 1, and 3 mg/L, and the selenium concentrations in the second row from left to right are 5, 10, 20, and 40 mg/L.

**FIGURE 3:**
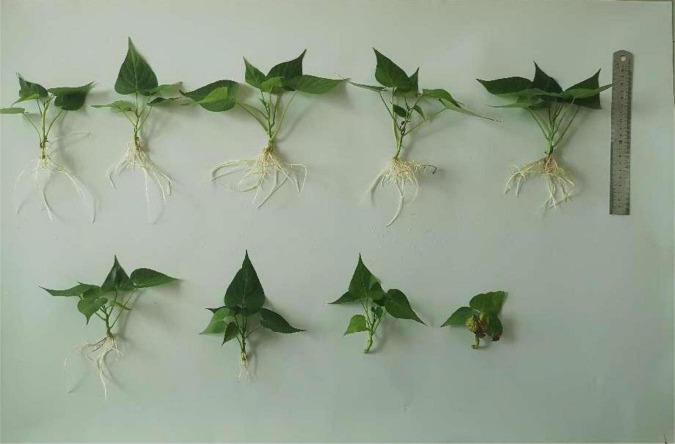
Growth of 20167-6 under different selenium concentrations. The selenium concentrations in the first row from left to right are 0, 0.2, 0.5, 1, and 3 mg/L, and the selenium concentrations in the second row from left to right are 5, 10, 20, and 40 mg/L.

**FIGURE 4:**
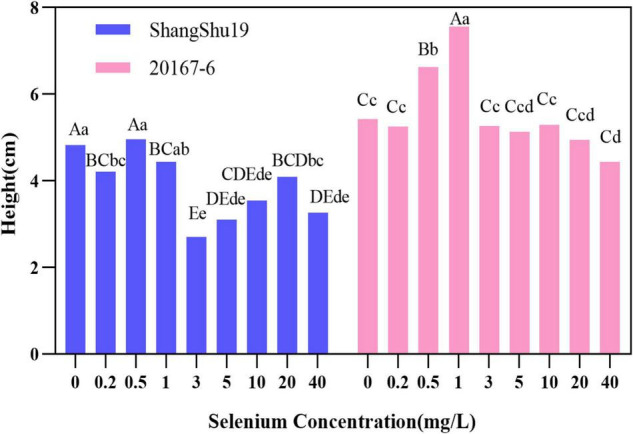
Effects of different selenium concentrations on the plant height of sweet potato. Different lowercase letters in the same category indicated that there were significant differences between Se treatments (Sig < 0.05); Different capital letters in the same category indicated that there were significant differences between Se treatments (Sig < 0.01).

There was an effect of selenium on plant height of sweet potato in water culture (Figures 2, 3). The plant height of Shangshu19 was higher (4.96 cm) than that of CK (4.83 cm) when the selenium concentration was higher than that of CK (0.5 mg/L). When the concentration of selenium exceeded 1 mg/L, the plant height was lower (2.70 cm) than that of CK, and the difference was very significant compared to CK, and the plant height of 3, 5, 10, and 40 mg/L was lower than that of 3.6 cm. When the selenium concentration of 0.5 and 1 mg/L was higher than that of CK, the plant height of 20167-6 was higher than that of CK, which was 6.62 and 7.56 cm, respectively. When the selenium concentration exceeded 1 mg/L, the plant height was lower than that of CK with the increase of selenium concentration. The plant height of 20 mg/L and 40 mg/L treatment was only 4.94 and 4.44 cm, and the plant growth was significantly inhibited (Figure 4 and Table 5).

**TABLE 5:** Effects of different selenium concentrations on the growth of sweet potato seedlings in water culture.

Variety	Selenium concentration (mg/L)	Height (cm)	Stem thick (cm)	Root length (cm)	Internode	Number of buckets	Fresh weight of stem and leaf (g)	Dry weight of stem and leaf (g)	Fresh weight of root (g)	Dry weight of root (g)	Stem and leaf dry fresh weight ratio (%)	Root dry fresh weight ratio (%)
Shangshu19	0	4.83Aa	3.34ABabc	10.80Aa	6.8Aa	7.8Aa	2.948Aa	0.24Aab	1.119Aa	0.058Aa	8.14Dd	5.21Bb
	0.2	4.22BCbc	4.06Aa	10.82Aa	6.6Aab	7.6Aab	2.192BCbc	0.19ABbc	0.909Bb	0.050Aa	8.55CDd	5.47ABb
	0.5	4.96ABa	3.45ABab	9.64ABb	6.4Aabc	7.4Aabc	2.671ABab	0.23Aab	1.035ABab	0.057Aa	8.68CDd	5.52ABb
	1	4.44ABCab	3.32ABabc	9.34Bb	5.9Abc	6.9Abc	2.243BCbc	0.19ABbc	0.882Cb	0.049Aa	8.67CDd	5.65ABb
	3	2.70Ee	3.27ABabc	6.37Cc	5.8ABc	6.8ABc	1.421Dd	0.15Bc	0.365CDc	0.027Bb	10.51BCc	7.51Aa
	5	3.11DEde	4.02Aa	5.61Cc	6.8Aa	7.8Aa	1.961CDc	0.20ABab	0.296DEc	0.023Bb	10.46BCc	7.47Aa
	10	3.54CDEcd	3.57ABab	2.02Dd	6.3Aabc	7.3Aabc	1.936CDc	0.22Aab	0.099Ed	0.008Cc	11.80ABbc	7.51Aa
	20	4.09BCDbc	2.62Bc	–	4.9BCd	5.9BCd	1.957CDc	0.26Aa	–	–	13.65Aa	–
	40	3.27DEde	3.20ABbc	–	4.6Cd	5.6Cd	1.903CDc	0.25Aa	–	–	13.13Aab	–
20167-6	0	5.43Cc	2.33Aa	12.44Aa	5.9Aab	6.8ABabc	1.171Dd	0.120Dd	0.599BCb	0.039Cc	10.14Dd	6.62BCc
	0.2	5.25Cc	1.87Ab	12.11ABab	5.4Ab	6.4ABbc	1.529CDcd	0.172CDc	0.809ABa	0.051ABCbc	11.28BCDcd	6.39Cc
	0.5	6.62Bb	2.11Aab	11.00ABCbc	5.7Aab	6.7ABabc	2.230ABab	0.237ABCabc	0.939Aa	0.062ABab	10.78CDd	6.67BCc
	1	7.56Aa	2.01Aab	10.64BCc	6.1Aa	7.1Aa	2.483Aa	0.266Aa	0.922Aa	0.064Aa	10.67CDd	6.96BCbc
	3	5.27Cc	2.14Aab	10.17Cc	5.7Aab	6.7ABabc	1.912BCbc	0.232ABCabc	0.573Cc	0.047BCc	12.17ABCbc	8.21ABCab
	5	5.14Ccd	2.22Aab	9.90Cc	5.9Aab	6.9ABab	1.902BCbc	0.246ABab	0.439Cc	0.041Cc	12.92ABab	9.20Aa
	10	5.30Cc	2.25Aab	2.99Dd	5.7Aab	6.7ABabc	1.852BCbc	0.233ABCabc	0.105Dd	0.010Dd	12.87ABab	8.37ABa
	20	4.94Ccd	2.20Aab	–	5.3Ab	6.2Bc	1.561CDcd	0.209ABCbcd	–	–	13.47Aab	–
	40	4.44Cd	1.97Aab	–	5.3Ab	6.3ABbc	1.384CDd	0.191BCbc	–	–	13.75Aa	–

*Different lowercase letters on the same column indicate a significant difference between Se treatments (Sig < 0.05). Different capital letters in the same column indicated that there were significant differences between Se treatments (Sig < 0.01).*

**FIGURE 5:**
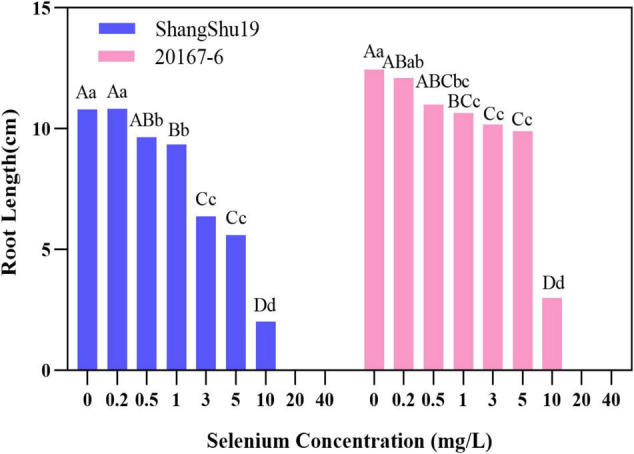
The effect of different selenium concentrations on the root length of sweet potato. The root length of sweet potato is 0 cm at the concentration of 20 and 40 mg/L selenium. Different lowercase letters in the same category indicated that there were significant differences between Se treatments (Sig < 0.05); Different capital letters in the same category indicated that there were significant differences between Se treatments (Sig < 0.01).

Selenium had a significant effect on the root length of sweet potato seedlings in water culture (Figures 2, 3). The increase of selenium concentration inhibited root growth of Shangshu19 and 20167-6, and root growth began to be inhibited when the selenium concentration was 3 mg/L, and root growth stopped under the treatment of 20 and 40 mg/L (Figure 5 and Table 5). Se concentration in 0.2 and 0.5 mg/L Shangshu19 decreased slightly compared with CK, and the decreasing trend was more prominent at 1 and 3 mg/L. With the increase in selenium concentration, the root dry weight of Shangshu19 was lower than that of CK, and there was significant difference in root dry weight compared with CK when selenium concentration was 3, 5, and 10 mg/L. Selenium concentration in 0.2, 0.5, 1, and 3 mg/L 20167-6. Stem and leaf dry weight, root dry weight increased compared to CK, in 0.5 mg/L, 1 mg/L increased more significantly, compared with the control, selenium concentration in 10 mg/L plant stem and leaf dry weight were significantly higher than the control, root dry weight were significantly lower than the control (Table 5). Compared with commercial potato Shangshu19, 20167-6, under the selenium concentration of 0.5, 1, and 3 mg/L, the plants grew more vigorously and had better selenium tolerance (Table 5).

### SPAD Value of Sweet Potato

**FIGURE 6:**
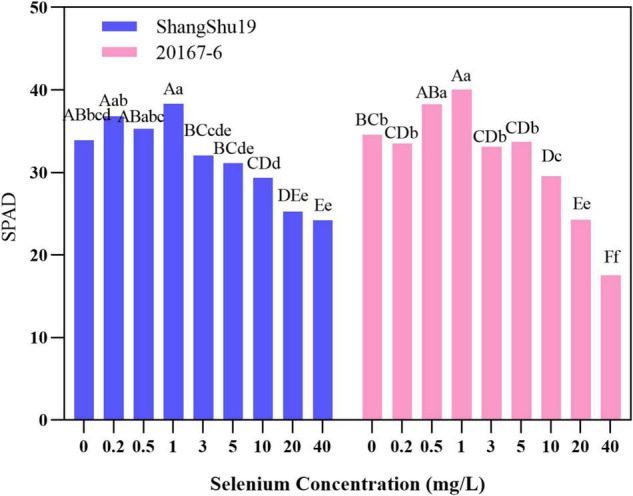
Effects of different selenium concentrations on SPAD value of sweet potato leaves. Different lowercase letters in the same category indicated that there were significant differences between Se treatments (Sig < 0.05); Different capital letters in the same category indicated that there were significant differences between Se treatments (Sig < 0.01).

Chlorophyll is the material basis of energy transformation in photosynthesis, and its content is a key index to measure leaf senescence and photosynthetic function. The relative content of chlorophyll in leaves can be determined by Nissan SPAD-502 Chlorophyll Meter. The leaf SPAD value of Shangshu19 was higher than that of CK under the selenium concentration of 0.2, 0.5, and 1 mg/L. The highest SPAD value of Shangshu19 was 38.32 under treatment of 1 mg/L selenium concentration. With increasing of selenium concentration, the SPAD value of Shangshu19 leaves gradually decreased. Under the 40 mg/L selenium concentration treatment, the lowest SPAD value of leaves was 24.18, significantly higher than that of CK, and there was a significant difference compared with the other five high concentration treatments. The leaf SPAD value of 20167-6 was higher than that of CK under the selenium concentration of 0.5 and 1 mg/L, and the highest SPAD value was 40.06 under the selenium concentration of 1 mg/L. With the increase of selenium concentration, the SPAD value of leaves gradually decreased. The difference of selenium concentration of 0.5 and 1 mg/L was significant compared to CK and 0.2 mg/L, and the difference was extremely significant compared with the other five high concentration treatments (Figure 6). This indicated that low concentration selenium (≤1 mg/L) treatment was helpful to increase the relative amount of chlorophyll in leaves of the tested materials, while high concentration selenium (≥3 mg/L) treatment began to be stressed to a certain extent, and the relative amount of chlorophyll in leaves decreased.

### Photoreaction Parameters of Sweet Potato Plants

**FIGURE 7:**
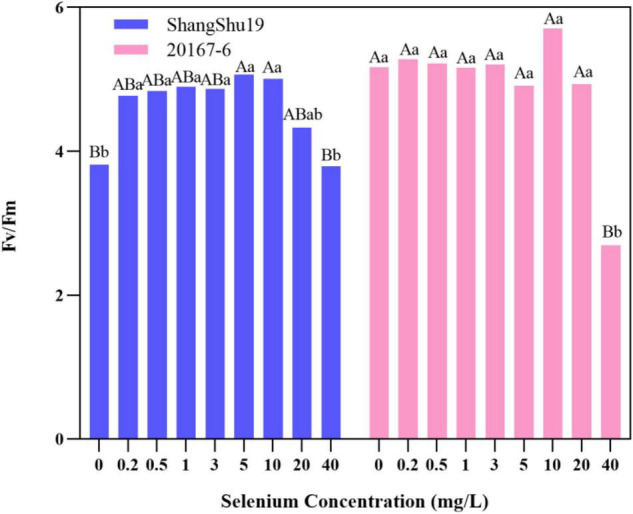
Effects of different selenium concentrations on the maximum photochemical efficiency (Fv/Fm) of PSII after dark treatment of sweet potato. Different lowercase letters in the same category indicated that there were significant differences between Se treatments (Sig < 0.05); Different capital letters in the same category indicated that there were significant differences between Se treatments (Sig < 0.01).

**FIGURE 8:**
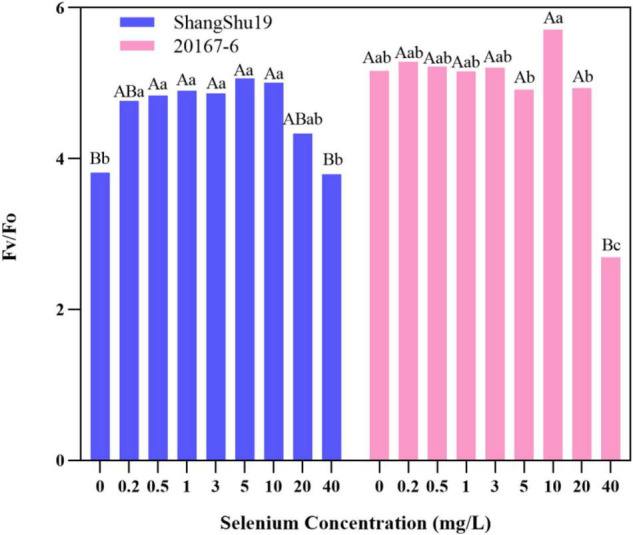
Effects of different selenium concentrations on potential activity (Fv/Fo) of PSII after dark treatment of sweet potato. Different lowercase letters in the same category indicated that there were significant differences between Se treatments (Sig < 0.05); Different capital letters in the same category indicated that there were significant differences between Se treatments (Sig < 0.01).

The maximum photochemical efficiency (Fv/Fm) of PSII in Shangshu19 leaves was significantly higher than that of CK when the selenium concentration was lower than that of 20 mg/L. At this time, the treatment of selenium concentration could significantly increase the opening degree and activity of PSII reaction center, and the opening degree and activity of Fv/Fm of leaves slightly lower than that of CK, PSII reaction center began to be inhibited when the selenium concentration was lower than that of 40 mg/L. 20167-6. When the selenium concentration of was lower than that of 5 mg/L 20167-6, the Fv/Fm value in leaves increased but the difference was not significant. At this time, the treatment of selenium concentration could increase the opening degree and activity of PSII reaction center. Under the concentration of 5, 10, 20, and 40 mg/L, leaf Fv/Fm opening and activity lower than that of CK began to inhibited PSII reaction center, and the value of Fv/Fm in leaves was lowest under the concentration of 40 mg/L selenium. Compared to CK, the difference is extremely significant (Figure 7). The potential activity (Fv/Fo) of PSII in leaves of Shangshu19 and 20167-6 at different selenium concentrations was consistent with the above-mentioned Fv/Fm (Figure 8). It is generally assumed that Fv/Fm under dark adaptation, is relatively constant under non-stress conditions, and is not affected by species and growth conditions. Under stress conditions, this parameter decreases significantly. Compared to 20167-6, Shangshu19 is less affected by selenium stress.

### Selenium Distribution in Sweet Potato Plants

**FIGURE 9:**
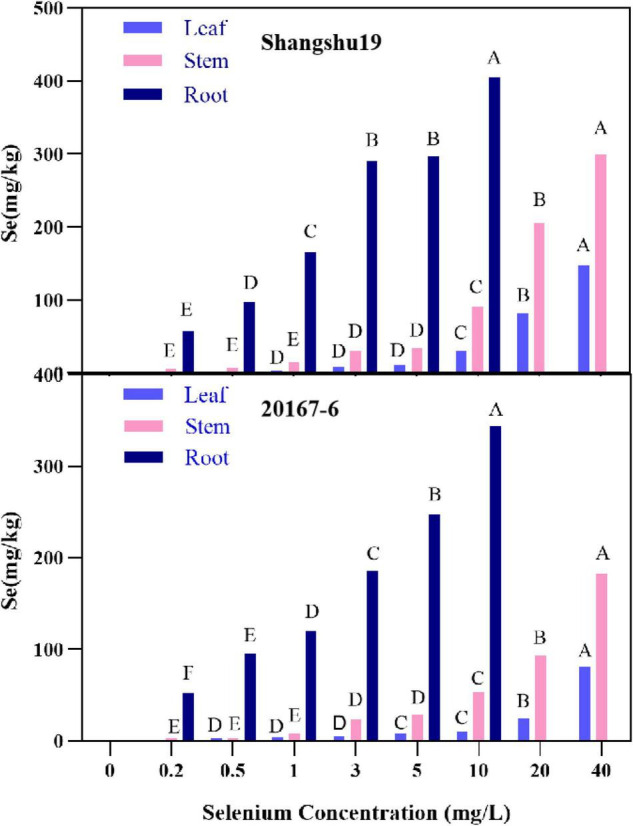
Effects of different selenium concentrations on the distribution of selenium content in Shangshu19 and 20167-6. Different capital letters in the same category indicated that there were significant differences between Se treatments (Sig < 0.01).

The selenium content in root, stem and leaf of Shangshu19 and 20167-6 increased with increasing of selenium concentration, and the highest selenium content in root system under 10 mg/L treatment was 405 and 344 mg/kg, respectively, significantly higher than that of other treatments. Under 40 mg/L treatment, the selenium content in stem and leaf of Shangshu19 and 20167-6 was the highest, which was 299, 148, 183, and 81.3 mg/kg, which was significantly higher than other treatments (Figure 9). The distribution of selenium content in plant was leaf <stem <root (Sig < 0.01), and the selenium content in root was significantly higher than that in stem and leaf. When the selenium concentration was higher than 1 mg/L, the accumulation of selenium in roots, stems and leaves of Shangshu19 was higher than 20167-6, and the plant was less affected by selenium stress.

## Discussion

### Effects of Se Concentrations on Sweet Potato Growth

In this study, a low Se concentration (≤1 mg/L) could increase plant height, which is consistent with the conclusion of Turakainen et al. (2004), but high Se concentration (≥3 mg/L) reduced plant height and root length, which was consistent with our conclusion that Akbulut and Çakır (2010) and Saffaryazdi et al. (2012) decreased plant height and root length with higher selenium concentration. When the concentration reached 20 mg/L, the root system of the plant stopped growing, but plant growth was not significantly affected until the selenium concentration increased to 40 mg/L. The high concentration may be toxic. Previously, selenium was widely used to mitigate oxidative damage in many plant species due to various environmental stresses (Xue et al., 2001; Pennanen et al., 2002; Turakainen et al., 2004; Germ et al., 2007). All of these results suggest that Se treatment can improve growth of many plants exposed to various environmental stressors, and this study also supports this conclusion. Here, we found that Se promoted the growth of 20167-6 sweet potato seedlings, and the plants treated with Se had higher dry matter weight of stems, leaves and roots than those without Se.

### Effects of Se Concentrations on Sweet Potato Photosynthesis

We found that low Se concentration increased the SPAD value of sweet potato seedling leaves, consistent with this study, low Se concentration increased the chlorophyll content of lettuce (Xue et al., 2001; Liu et al., 2022). Hawrylak-Nowak (2009) reported that the increase in chlorophyll content due to the effective scavenging of reactive oxygen species (ROS) caused by the addition of Se. Low Se concentration can enhance photosynthetic pigment biosynthesis (Pennanen et al., 2002) by protecting chloroplast enzymes and enhancing the photosynthetic pigments biosynthesis. Plant photosynthetic physiology is particularly sensitive to environmental stress, and selenium can regulate environmental stress and may further affect plant photosynthesis. Freeman et al. (2010) compared the electron transfer rate [electron transport rate (ETR)] of –selenium rich plants and non-selenium-rich plants under selenium treatment, and found that 20 μmol selenate treatment of selenium rich plants significantly increased their ETR, while non-selenium rich plants showed the opposite under the same conditions. Application of selenium (<50 g/ha) increased photosynthetic rate (Pn), ETR photosynthesis index and chlorophyll fluorescence parameters such as Fv, Fo, Fv/Fm, and Fv/Fo in rice. When the selenium application rate was 100 g/ha, the photosynthetic index decreased (Zhang et al., 2014). We found that the values of Fv/Fm and Fv/Fo of plants treated with selenium concentration of 40 mg/L decreased compared to CK, and the photosynthesis of plants was inhibited, consistent with the results of Zhang et al. (2014). However, we only examined the changes of SPAD value, Fv/Fm and Fv/Fo value of Shangshu19 and 20167-6 under selenium concentration treatment, which need to be further investigated from the aspects of physiological level and broader genetic molecular mechanism.

### Effects of Se Concentrations on Sweet Potato Selenium Content Distribution

The fact that selenium application increased the Se concentration in potato plants was proportional to the addition level, which indicated that the added sodium selenate was effectively utilized (Turakainen et al., 2004). Products with selenium fertilizer applied to the soil or foliage can effectively increase selenium content (Broadley et al., 2006; White and Broadley, 2009; Chilimba et al., 2012; Fordyce, 2013; Alfthan et al., 2015). Agricultural products planted on selenium soil had higher selenium availability in plants (Ihnat, 1989; Broadley et al., 2006; Williams et al., 2009; Lee et al., 2011; Garrett et al., 2013; Joy et al., 2015; Liu et al., 2020a,b). In fact, application of inorganic selenium fertilizers is particularly effective in increasing selenium content in edible crops, increasing selenium content in animal feed, and improving selenium status and health in animals and humans (White and Broadley, 2009; Alfthan et al., 2015; Liu et al., 2021). We found that selenium application significantly increased the Se concentration in upper leaves, stems and roots, and the distribution of selenium content in plants was leaf <stem <root. Plant roots obtain selenium from rhizosphere solution, and root cells can absorb selenite, selenite and organic selenium compounds (König et al., 2012; Liu et al., 2020c). After selenium fertilizer was absorbed by plant root cells, selenite quickly moved to the stele through the symbiote of root and was transferred to the shoot, while selenite was converted into organic selenium compounds, that normally remained in the root (Li et al., 2008; Wang et al., 2015; Huang et al., 2017).

The selenium content in young leaves of plants is the highest, which generally peaking during seedling growth, and then decreasing before or during flowering, as selenium is transferred from the leaves to reproductive organs (Turakainen et al., 2004; Galeas et al., 2007; Cappa et al., 2014; Harris et al., 2014; Yan et al., 2022). When the selenium concentration was higher than 1 mg/L, the accumulation of selenium in roots, stems and leaves of Shangshu19 was higher than 20167-6, and the Fv/Fm content decreased less than CK, and was less affected by selenium stress. In summary, we think that low selenium concentration is beneficial to the growth of sweet potato seedlings and the accumulation of dry matter content and selenium content in each tissue.

## Conclusion

Treatment with a selenium concentration of 1 mg/L was helpful to increase leaf SPAD and plant height. At 3 mg/L, leaf SPAD gradually decreased with increasing of selenium concentration, plant root growth began to be inhibited, plant Fv/Fm and Fv/Fo decreased compared to CK under 40 mg/L selenium concentration treatment, plant photosynthesis was inhibited, and root growth stopped. The selenium content in root, stem and leaf increased with the increase of selenium concentration, and the distribution of selenium content in plant was leaf <stem <root. In summary, the suitable concentration of selenium tolerance of virus-free sweet potato seedlings in water culture was 3 mg/L.

## Data Availability Statement

The original contributions presented in the study are included in the article/supplementary material, further inquiries can be directed to the corresponding author/s.

## Author Contributions

HC, YX, and YS initiated and designed the research. HC and QuC performed the experiments. QiC, XY, YQ, WS, SF, JG, and SS revised and edited the manuscript, and provided advice on the experiments. All authors contributed to the article and approved the submitted version.

## Conflict of Interest

The authors declare that the research was conducted in the absence of any commercial or financial relationships that could be construed as a potential conflict of interest.

## Publisher’s Note

All claims expressed in this article are solely those of the authors and do not necessarily represent those of their affiliated organizations, or those of the publisher, the editors and the reviewers. Any product that may be evaluated in this article, or claim that may be made by its manufacturer, is not guaranteed or endorsed by the publisher.
